# Real World Data in Health Technology Assessment of Complex Health Technologies

**DOI:** 10.3389/fphar.2022.837302

**Published:** 2022-02-10

**Authors:** Milou A. Hogervorst, Johan Pontén, Rick A. Vreman, Aukje K. Mantel-Teeuwisse, Wim G. Goettsch

**Affiliations:** ^1^ Division of Pharmacoepidemiology and Clinical Pharmacology, Utrecht Institute for Pharmaceutical Sciences (UIPS), Utrecht University, Utrecht, Netherlands; ^2^ National Health Care Institute (ZIN), Diemen, Netherlands; ^3^ The Dental and Pharmaceutical Benefits Agency (TLV), Stockholm, Sweden

**Keywords:** real-world data, health technology assessment, complex health technologies, Europe, questionnaire

## Abstract

The available evidence on relative effectiveness and risks of new health technologies is often limited at the time of health technology assessment (HTA). Additionally, a wide variety in real-world data (RWD) policies exist among HTA organizations. This study assessed which challenges, related to the increasingly complex nature of new health technologies, make the acceptance of RWD most likely. A questionnaire was disseminated among 33 EUnetHTA member HTA organizations. The questions focused on accepted data sources, circumstances that allowed for RWD acceptance and barriers to acceptance. The questionnaire was validated and tested for reliability by an expert panel, and pilot-tested before dissemination *via* LimeSurvey. Twenty-two HTA organizations completed the questionnaire (67%). All reported accepting randomized clinical trials. The most accepted RWD source were patient registries (19/22, 86%), the least accepted were editorials and expert opinions (8/22, 36%). With orphan treatments or companion diagnostics, organizations tended to be most likely to accept RWD sources, 4.3–3.2 on a 5-point Likert scale, respectively. Additional circumstances were reported to accept RWD (e.g., a high disease burden). The two most important barriers to accepting RWD were lacking necessary RWD sources and existing policy structures. European HTA organizations seem positive toward the (wider) use of RWD in HTA of complex therapies. Expanding the use of patient registries could be potentially useful, as a large share of the organizations already accepts this source. However, many barriers still exist to the widespread use of RWD. Our results can be used to prioritize circumstances in which RWD might be accepted.

## Introduction

Sufficient amounts and quality of data on a treatment’s effects, safety and costs is of crucial importance in order to minimize uncertainty in decision-making on reimbursement (Vreman et al., 2020a; Hogervorst et al., 2021). However, the available evidence for these evaluations can be limited (Hogervorst et al., 2021). Novel, complex treatment strategies such as health technologies with concomitant genetic testing, advanced therapy medicinal products (ATMPs) or the assessment of sequences of treatments with disease-modifying capacities may be especially prone to these limitations (Garrison et al., 2015; Lipska et al., 2015; Berm et al., 2016; Vreman et al., 2019; Ghabri et al., 2020; Lloyd-Williams and Hughes, 2020). A survey among European health technology assessment (HTA) organizations assessed challenges associated with such complex health technologies. HTA assessors reported various challenges occurring at all steps throughout the HTA process, but regardless of step in the process, most of the reported challenges rooted in data insufficiencies at the time of HTA assessment (Hogervorst et al., 2021).

Some of these complex health technologies inherently cause data insufficiencies, making them more challenging for HTA (Ribeiro et al., 2020). Often there is uncertainty around long-term claims for gene-therapies and other ATMPs as clinical trials do not cover life-long effects (Fruergaard Jorgensen, 2017; Jönsson et al., 2019; Coyle et al., 2020). There is an increasing amount of precision medicines and orphan medicines that may limit the possibility of performing well-controlled, large trials (Margaret, 2013; Moloney et al., 2015; Griffiths et al., 2017). Additionally, patients’ access to new health technologies may be requested more early in the authorization and reimbursement process because of high unmet medical needs, which has led to the development of expedited approval processes (Kesselheim et al., 2015; Leyens and Brand, 2016). This trend also results in decreasing amounts and quality of data available at the time of the HTA process. For example, interim data or effectiveness data only based on surrogate endpoints is all that is available upon approval of a newly developed intervention (Larson et al., 2017; Pruce et al., 2017; Blagden et al., 2020; Ribeiro et al., 2020).

For both reasons -increased complexity of health technologies and expedited approval processes-there is an increased necessity of real-world data (RWD) as an addition to data from traditional randomized controlled trials (RCT) (Makady et al., 2017a; IMI GetReal, 2021). Simultaneously, there is a strong voice supporting the use of “real-world” effectiveness in addition to the RCT-tested efficacy for reimbursement decision-making, due to another limitation of RCTs referred to as the “efficacy-effectiveness gap”. (Eichler et al., 2011; Ankarfeldt et al., 2017).

Published economic evaluations based on RWD, on the other hand, have trouble meeting quality criteria, as guided by the CHEERS checklist, for properly accounting for biases (Guertin et al., 2017; Parody-Rúa et al., 2020). In general, the quality in terms of internal validity is a concern with RWD, which has been extensively discussed (Collins et al., 2020; Eichler et al., 2020; Ramagopalan et al., 2020). The lack of external validity of RCTs and current quality issues with RWD make reimbursement decisions difficult.

As the implementation of RWD in HTA is challenging but nevertheless increasing, organizations such as the Professional Society for Health Economics and Outcomes Research (ISPOR), the International Society for Pharmacoepidemiology (ISPE), European Network for HTA (EUnetHTA, or EUnetHTA 21), the Food and Drug Administration (FDA) and European Medicines Agency (EMA) have published their positions on inclusion of RWD in regulation and HTA decision-making (Berger et al., 2017; Public Policy Committee and International Society of Pharmacoepidemiology, 2016; Research C for DE, 2020; Anonymous, 2018; EUnetHTA Publication of a JA2 methodological guideline, 2015; ENCePP Home Page, 2021). These statements generally agree that communication, for example on study protocols, outcomes selection, measurement, reporting and interpretation is important as well as stakeholder collaboration or alignment regarding the design, conduct and interpretation of RWD studies. All statements describe that careful consideration is needed for each individual situation to assess whether the use of RWD is appropriate, focusing on internal validity (various forms of bias) or external validity (applicability to practice), and some even indicate the methods that should be used.

Makady et al. demonstrated, with large differences among HTA organizations and between all the domains of the HTA process, that current policies and guidelines from HTA agencies in six European countries did in general not actively encourage the use of RWD (Makady et al., 2017a). Certainly this was the case in relative effectiveness assessments (REAs), whereas the interest in RWD in the case of cost-effectiveness assessments (CEAs) was wider, sometimes even requested. In HTA practice of oncological drugs for the treatment of melanoma, another study by Makady et al. showed that in five European countries, the actual use of RWD was indeed higher for CEA parameters than for the REA, although many differences among the countries were still observed (Makady et al., 2018). Alignment on policies for RWD use could increase the adaptation of RWD in HTA processes in Europe.

Literature is not conclusive on the types of RWD that are accepted, nor on the transferability of these results to other countries. Additionally, there is a lack of knowledge about the circumstances in which RWD is more likely to be used, such as specific types of complex health technologies or procedural circumstances. Besides the general concern on quality and robustness of RWD, there is no insight in barriers to the use of RWD in HTA. In an attempt to guide future research on method development and alignment of policies for expanding use of RWD in HTA, *this study assesses which challenges in HTA, related to the increasingly complex nature of new health technologies, make the acceptance of RWD most likely, based on practical experiences by European HTA organizations*.

## Materials and Methods

Data were collected through dissemination of a questionnaire. This strategy allowed us to gain insight in daily practice in HTA organizations. The questions on RWD were combined with questions informing additional deliverables in the HTx project, all focusing on complex treatments. The HTx project is a Horizon 2020 project supported by the European Union lasting for 5 years from January 2019, with the aim to create a framework for the Next Generation HTA to support patient-centered, societally oriented, real-time decision-making on access to and reimbursement for health technologies throughout Europe (HTx, 2020). The broad definition for RWD that we used was ‘all routinely collected data on patients that are not RCTs’ (Makady et al., 2017b). A full description of the methods on the questionnaire’s development, validation and dissemination is published in an earlier study (Hogervorst et al., 2021).

Both national and regional European HTA organizations were invited using the EUnetHTA member database, 33 in total. This approach ensured the representation of a balanced mixture of European countries. All organizations were directly involved in decision-making or advised decision-making parties. The targeted representatives in these organizations were experienced HTA assessors (at least 3–5 years of experience), to ensure sufficient knowledge and experience. Appendix 1 shows a list with all selected and responding HTA organizations.

### Questionnaire Structure

Three topics were addressed in the part of the questionnaire that focused on RWD: the general willingness to use and accept RWD, likelihood to accept RWD in particular challenging circumstances and the barriers to accept RWD. First, respondents could indicate whether they experienced the need for wider systematic use of RWD and whether they experienced a willingness among assessors, among decision-makers or among both groups, as both affect the ultimate reimbursement decision. Second, respondents selected in a binary way (yes/no) the types of data sources that were accepted for assessment by their organization. Third, respondents indicated their likelihood for accepting RWD sources in predefined challenging circumstances on a 5-point Likert scale. The selection of these predefined treatments has been described in an earlier study (Hogervorst et al., 2021). In the fourth and only open question, respondents could describe additional circumstances that they encountered that would allow for the acceptance of RWD in HTA, ensuring the sensitivity of the questionnaire. Last, the respondents ranked a list of barriers to accepting RWD for HTA. This list was based on literature and practical experience of authors (JP, RV, WG) affiliated at HTA organizations.

### Questionnaire Validation and Testing

The questionnaire was thoroughly tested for validation and reliability before dissemination (Kimberlin and Winterstein, 2008; Bolarinwa, 2020; Venkitachalam, 2021). An expert panel with two representatives from academia and five from three HTA organizations (i.e. the National Institute for Health and Care Excellence [NICE], the Dental and Pharmaceutical Benefits Agency [TLV], the Dutch National Health Care Institute [ZIN]), tested the questionnaire for content and face validity, as well as reliability. Subsequently, a pilot test was performed to test the feasibility of completion and correct interpretation of the full set of questions. The pilot test was not considered in the presented results and performed by other representatives in the specific HTA organizations than the final participants. The more detailed development and validation approach has also been described in earlier published work (Hogervorst et al., 2021).

### Dissemination and Analysis

The questionnaire was built in LimeSurvey (LimeSurvey GmBH, Hamburg, Germany) and was disseminated between January and February 2020 (Schmitz, 2020). To increase the response rate, an announcement as well as 3 reminders were sent to targeted participants. The analyses were performed in Microsoft Excel (Microsoft, Redmond, WA) and GraphPad Prism v9.0.0 for Windows (PraphPad Software, San Diego, CA) (Microsoft Excel and Spreadsheet Software, 2020; Home - GraphPad [Internet], 2021). Descriptive statistics were used to visualize the questionnaire results, using averages, bar charts, boxplots and tables for the open question.

## Results

Out of 33 invited HTA organizations, 22 organizations from 21 different countries completed the questionnaire (response rate 67%), see appendix. There was a relatively balanced spread of organizations throughout Europe, with a slight overrepresentation from the Nordic countries. Twenty-one responding organizations (95%) were responsible for assessing pharmaceuticals, of which nine (41%) were assessing solely pharmaceuticals. Thirteen organizations (59%) were responsible for assessment of non-pharmaceuticals, of which one (5%) solely assessed non-pharmaceuticals. Consequently, twelve organizations (55%) were responsible for assessing both pharmaceuticals and non-pharmaceuticals.

### Willingness to Use and Accept RWD Sources in HTA

**FIGURE 1:**
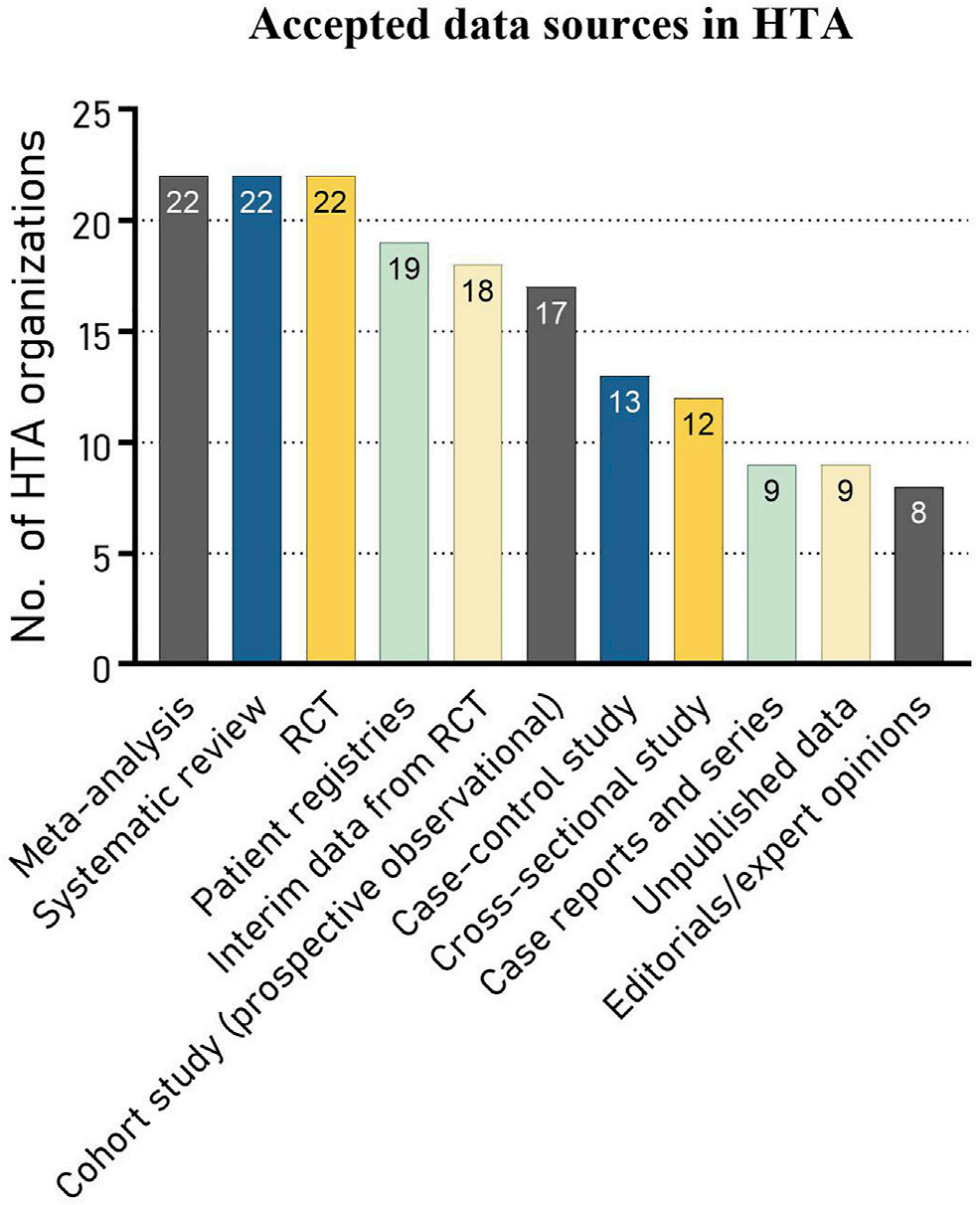
Different data sources accepted by HTA organizations (N = 22).

Out of 22 representatives, 18 (82%) indicated to see a need for wider systematic use of RWD in HTA decisions than is currently in practice. Sixteen representatives indicated that they experience a willingness among both assessors and decision-makers, two representatives indicated they experience a willingness only among the assessors and one only among decision-makers. The remainder indicated to have no knowledge about the willingness or to experience no willingness at all. When looking at the accepted data sources, the traditional sources, i.e., meta-analyses, systematic reviews and randomized controlled trials (RCTs), embodied the top three accepted data sources for HTA in Europe. All three were accepted by all participating organization. This was followed by patient registries, which were accepted by 19 HTA organizations (86%). Case reports, unpublished data and editorial and expert opinions were among the least accepted RWD sources, each accepted by one third of the organizations (Figure 1).

### Likelihood to Accept RWD in Challenging Circumstances

**FIGURE 2:**
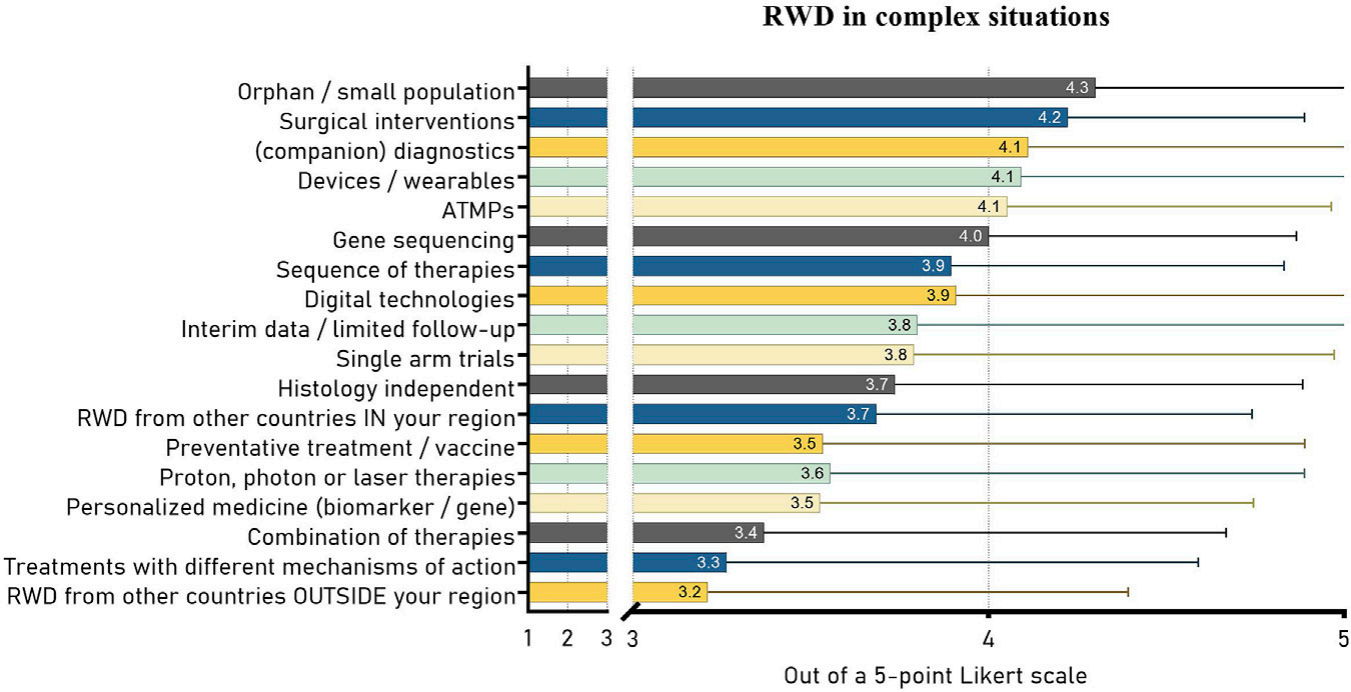
Average Likert scores (1–5) with standard deviation of the likelihood to accept (additional) RWD in various challenging circumstances in HTA. ATMP, advanced therapy medicinal product.

The assessment of orphan drugs or other treatments with small patient populations was presented as the most likely situation to accept RWD sources, scoring 4.3 out of the 5-point Likert scale. This was closely followed by (companion) diagnostic procedures and surgical interventions, scoring 4.2 and 4.1, respectively. Organizations would be least likely to accept RWD in HTA if this data came from countries in regions outside their own region, despite it being the only available data source (3.2), see Figure 2. The scores ranged between 3.2 and 4.3, creating a substantial gap in likelihood of accepting RWD between the first and last ranked situation (between orphan drugs and using RWD from outside your country’s region). Generally, the results indicate that among these circumstances the attitude towards RWD acceptance leans more toward positive than to negative.

Seven organizations reported additional circumstances where RWD may be accepted. Some reported that RWD would not be accepted as the sole source of evidence, though could be supplementary to traditional RCT evidence. In case of no available RCT data, single-arm studies could be accepted. However, a high level of uncertainty would still be a concern in this case. Table 1 shows all reported circumstances in which organizations would be willing to accept RWD, as derived from the open question.

**TABLE 1:** Additional circumstances in which the HTA organizations would be willing to accept RWD for their assessments. RWD, real world data; RCT, randomized clinical trial; CEA, cost-effectiveness analysis; HT, health technologies.

*CATEGORY ARGUMENT*	*ARGUMENT*
*POPULATION*	RWD would be accepted in case of a high burden of disease if the indications of the assessed treatment are very severe or even fatal
*INTERVENTION*	RWD would be used in case of highly innovative HTs which are just approaching marketing readiness. Additionally, if the treatment would otherwise not be available or accessible
*COMPARATOR*	Where the trials used for licensing compare against treatments that are not used in the country’s practice, which is similar to issue with single arm trials, RWD would be accepted
*OUTCOME*	RWD would be used in case of lacking robust evidence, however, if the treatment does suggests highly promising results based on the (not robust) literature that is available RWD would be accepted when the findings of the RCT are outdated, or in case of considerable contradictories in the available RCT literature RWD are more likely to be used where the data has potential to resolve areas of uncertainty in the clinical case
*CEA*	In case of considerably high uncertainties in the cost-effectiveness analysis, RWD would be used to feed into the assessment
*POLICY*	RWD would be used in case of pharmaceuticals that are authorized under the European WEU^1^ legislation, because this approval is inherently based on RWD. Additionally, in the case of interventions requiring informed consent schemes
*PRACTICE*	In the case where there is uncertainty over resource utilization in clinical practice

^1^Well-established use: this is the case if the active compound in a pharmaceutical has been used for more than 10 years and the efficacy and safety are thus “well-established”. WEU product dossiers need to fulfil legislative requirements of Directive 2001/83/EC by showing that the product applying for market access is safe and efficacious and of high quality

### Barriers to Accept RWD

**FIGURE 3:**
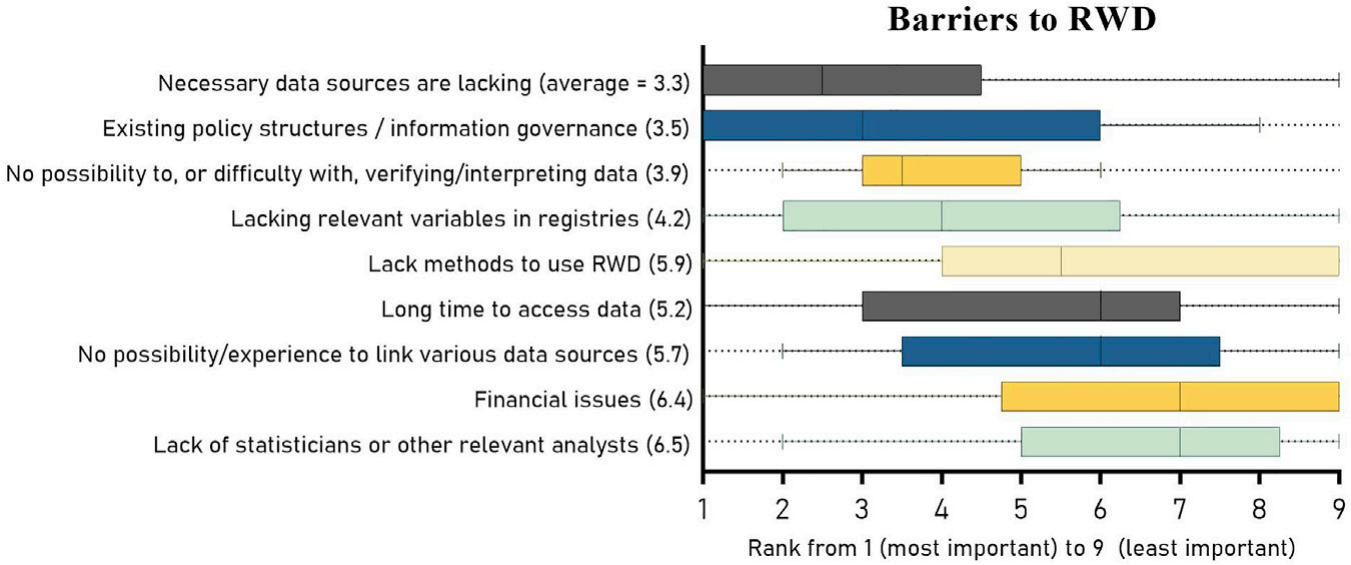
Boxplots of the ranking of the barriers to accepting RWD in HTA or decision-making. The barriers are placed in order of their median ranked score and include the 25th and 75th percentile as well as the minimum and maximum rank. The mean scores are listed between brackets on the left side.

There was considerable variation in responses of HTA agencies. On average, the organizations ranked “lacking necessary RWD sources” as the most important barrier to being able to accept RWD in HTA with a mean rank of 3.3, see Figure 3. This was followed by “existing policy structures or information governance” (mean rank 3.5) that complicated accepting RWD, and third, that there was “no possibility to interpret or verify data, or that it was challenging to do so” (mean rank 3.9). “Financial reasons” and “lack of statisticians or other relevant analysts” ranked last. When considering the median in the boxplots instead of the mean ranks listed on the left side of the figure, the order of ranking is almost equal, except for “lack methods to use RWD”, due to a skewed spread of rankings (Figure 3). “No possibility to interpret or verify data, or that it was challenging to do so” showed the most consistent ranking among all reasons (smallest interquartile range). Additionally, the medians of “necessary data sources are lacking’ (2.5) and “existing policy structures or information governance” (3.0) show that, despite the wide range of ranks, more than half the HTA organizations ranked these two reasons in the top three.

## Discussion

As input for their HTA decision-making process, all HTA organizations reported the acceptance of meta-analyses, systematic reviews and traditional RCTs. As expected, data sources not based on RCTs were less accepted. Patient registries were the most accepted RWD source. The least accepted sources–accepted by only one third—included case series or reports, unpublished data sources and editorials or expert opinions. A large share of respondents indicated to experience the need and willingness among both assessors and decision-makers for wider systematic use of RWD in HTA. HTA assessors also indicated a likelihood to accept RWD sources in all of our pre-defined “complex” situations, as well as in a number of additionally reported situations. Among predefined barriers to using RWD, lacking sources and existing policy structures ranked highest. Despite the great spread in answers among HTA organizations, lacking resources (available methods, sufficient finances or skilled employees) seemed to be least hindering the use of RWD.

Makady et al. found in RWD policies among six European HTA organizations, that all six accepted any source of data, including RWD, albeit with a certain hierarchy (22). Based on our results from 21 countries, it seems that other European countries may be more reluctant to accept RWD. The hierarchy among data sources is, however, also reflected in our results. Makady’s finding that registries are widely used seem to be transferable to other European countries based on our results (Makady et al., 2018). Moreover, their study showed that RWD was uncommonly accepted in the initial assessment of effectiveness, however for an estimation for long-term effectiveness (stretching further than RCT length) RWD seemed to become more relevant (Makady et al., 2018). As long-term effect claims based on short RCTs are a common feature in ATMP assessment, this explains why ATMPs ranked in the top 5 circumstances to accept RWD in the present study.

According to our questionnaire, difficulty with verifying and interpreting RWD seemed to be a less important barrier to the use of RWD than the lack of data and existing policies. However, an inquiry by Facey et al., indicated that a lack of clarity on methods to assess real-world evidence (RWE) is seen by HTA agencies as a major obstacle to adequate use of RWE in HTA (Facey et al., 2020). Our ranking method solely visualized the order of importance. Combined with these previous findings we may conclude that all of our listed barriers, including the lowest ranked, should be considered as important. Therefore, initiatives to help overcome barriers to use RWD in HTA are an important next step. These initiatives could first include methodological work on RWD quality assessment, alternative trial designs or combining RCT with RWD sources as well as implementing methods into the decision making process (Selker et al., 2014; Makady et al., 2017c; Efthimiou et al., 2017; Selker et al., 2019; Chalkou et al., 2021). Second, the focus could be on consensus building on when and how to use RWD (methods) (Berger et al., 2017; Vreman et al., 2020b; Facey et al., 2020; Orsini et al., 2020). A third suggestion could be to invest more in generating and aligning the required data, such as patient registries, at an early stage, as is aimed by the European IMI EHDEN project as well as by national initiatives across the continent (Ministerie van VolksgezondheidWen, 2019; IMI Innovative Medicines Initiative EHDEN, 2021; The National Patient Register, 2021).

Our results, supported by existing initiatives, could be used to guide prioritization. Reasoning from the most likely situations where RWD may be acceptable based on our findings, focus could first be put on RWD for orphan drugs, ATMPs, data for assessments of sequences of treatments, promising treatments for high burden diseases or other treatments that generally come with high uncertainty (Makady et al., 2017a; Didden et al., 2018; Moseley et al., 2020; Hogervorst et al., 2021). Non-pharmaceuticals, including surgical interventions, diagnostics, wearables or other devices, gene sequencing techniques and digital interventions, need special attention as these do not generally require the same data quality standards as compared to pharmaceuticals (FITTH, 2018). Unfortunately, due to a low number of agencies assessing non-pharmaceuticals, we could not compare responses between agencies that assess medicines only versus those that also or only assess non-pharmaceuticals in the present study.

Due to new European HTA legislation, a new consortium of HTA partners called EUnetHTA21, will provide an updated methodological framework (Health Technology Assessment, 2021; History of EUnetHTA, 2021). At this moment it is still unclear how questions on collection, use and assessment of RWD will be addressed in the activities of EUnetHTA21. However, the current divergence in HTA policies and RWD acceptance found in literature (Makady et al., 2017a; Makady et al., 2018; Health Technology Assessment, 2021), as well as our results indicating that policies still complicate the use of RWD, suggest that at least European coordination and consensus-building is necessary to ensure adequate, consistent and reliable use of RWD in HTA practice.

### Strengths and Limitations

In this study we used the term RWD as referring to all routinely collected information about people outside a randomized controlled trial setting, and RWE as referring to the evidence derived from the analysis of RWD (Makady et al., 2017b; Gonzalez, 2020; Commissioner O of the. Real-World Evidence, 2021). We acknowledge the discussion surrounding the terminology, as many new, flexible, adaptive and pragmatic trial designs emerge, even in registries, making it more difficult to distinguish between a controlled setting and uncontrolled setting (Efthimiou et al., 2017; Didden et al., 2018; Schünemann, 2019; Ford and Norrie, 2016; Pallmann et al., 2018; Li et al., 2016; Hamers et al., 20215). RWD, however, is a widely recognized term, specifically among our targeted participants.

Our results were informed by responses from 22 HTA organizations representing 21 European countries. Only a few larger countries such as Portugal, France and Italy did not respond. We would, however, not expect large differences since we included a large cohort of countries, small and large, from all European regions. Additionally, the disseminated questionnaire was well tested for validity and reliability by an expert panel, including experienced representatives from HTA practice. This ensures our questions and thus results are well aligned with practice. The inclusion of multiple middle/lower income countries in Europe allows for some transferability of results to similar countries in other regions that perform HTA and wish to accept (more) RWD. (Justo et al., 2019; Lou et al., 2020).

We did not include the newer advanced (pragmatic or flexible) trial designs in our questionnaire, so we do not know yet how these relate to observational designs. However, as these designs are new and not yet widely used, it is likely that HTA organizations have not encountered them often. Inquiring after the differences between initial assessments an reassessments and additional barriers may be useful, but were outside the scope of this study.

## Conclusion

Despite the wide variety in acceptance of RWD sources and in the concomitant policies, assessors from European HTA organizations do seem to be positive toward the (wider) use of RWD for their assessments. Expanding use of patient registries seems potentially useful as a large share of organizations already accept this data source. Our results can be used to prioritize health technologies and circumstances in which accepting RWD could be desired. Initial focus could be placed on orphan therapies and diagnostic tools, innovative treatments for high burden diseases or on health technologies that generally come with high uncertainty. However, many barriers to the use of RWD still exist. Future research and policies should focus on strategies to build and maintain high quality patient registries as well as on consensus building and implementation of trustworthy evidence generation and assessment methods based on these registries.

## Data Availability

The raw data supporting the conclusions of this article will be made available by the authors, without undue reservation.

## Footnotes

1 Well-established use: this is the case if the active compound in a pharmaceutical has been used for more than 10 years and the efficacy and safety are thus “well-established”. WEU product dossiers need to fulfil legislative requirements of Directive 2001/83/EC by showing that the product applying for market access is safe and efficacious and of high quality
