# Supplementary material for: Real World Data in Health Technology Assessment of Complex Health Technologies
Source: Front Pharmacol. 2022 Feb 10;13:837302. doi: 10.3389/fphar.2022.837302 (PMC8866967; doi:10.3389/fphar.2022.837302)
Supplement: Supplementary file 1 [file Table1.docx]

Table 1 Organizations in European countries invited to fill in the questionnaire. In bold are the organizations that completed the questionnaire.

| **Country** | **Organisation** |
| --- | --- |
| **Austria** | **Austrian Social Insurance (HBV)** |
| **België** | **"Rijksinstituut voor Ziekte- en Invaliditeitsverzekering" (RIZIV INAMI)** |
| **Bulgaria** | **National Center of Public Health and Anlayses (NCPHA)** |
| **Croatia** | **Ministry of Health (MoH)** |
| Cyprus | Ministry of Health |
| **Czech Republic** | **State Institute for Drug Control (SUKL)** |
| **Denmark** | **Danish Medicines Council (DMA)** |
| **Finland** | **Finnish Medicines Agency (FIMEA)** |
| France | “Haute Autorité de Santé” (HAS) |
| **Germany** | **Gemeinsamer Bundesausschluss (B-GA)** |
| **Hungary** | **National Institute of Pharmacy and Nutrition (NIPN)** |
| Hungary | Department of Technology Assessment |
| **Ireland** | **National Centre for Pharmacoeconomics (NCPE)** |
| Italy | The Italian Medicines Agency (AIFA) |
| Lithuania | State Health Care Accreditation Agency under the Ministry of Heatlh of the Republic of Lithuania (VASPVT) |
| **Malta** | **Directorate for Pharmaceutical Affairs, Ministry for Health Malta (DPA/MFH)** |
| **Netherlands** | **The Dutch National Health Care Institute (ZIN)** |
| **Norway** | **Norwegian Medicines Agency (NOMA)** |
| **Poland** | **Agency for Health Technology Assessment and Tariff System (AOTMiT)** |
| Portugal | National Authority of Medicines and Health Products (INFARMED) |
| **Romania** | **National School of Public Health, Management and Professional Development Bucharest (SNSMPS)** |
| **Scotland** | **Scottish Health Technologies Group (SHTG)** |
| **Scotland** | **Scottish Medicines Consortium (SMC)** |
| **Slovakia** | **Comenius University in Bratislava Faculty of Pharmacy (UNIBA-FoF)** |
| Slovakia | Ministry of Health |
| Slovenia | Agency for Medicinal Products and Medical Devices of the Republic of Slovenia (JAZMP) |
| Slovenia | Ministry of Health |
| **Spain** | **Basque Office for Health Technology Assessment (OSTEBA)** |
| Spain | The Spanish Agency of Medicines and Medical Devices (AEMPS) |
| Spain | Galician Agency for Health Technology Assessment (AVALIA-T) |
| **Sweden** | **The Dental and Pharmaceutical Benefits Agency (TLV)** |
| **Switzerland** | **Federal Office of Public Health (FOPH - BAG)** |
| **United Kingdom** | **the National Institute for Health and Care Excellence (NICE)** |
